# Machine learning models for outcome prediction in thrombectomy for large anterior vessel occlusion

**DOI:** 10.1002/acn3.52185

**Published:** 2024-08-23

**Authors:** Omid Shirvani, Stefanie Warnat‐Herresthal, Ivan Savchuk, Felix J. Bode, Louisa Nitsch, Sebastian Stösser, Taraneh Ebrahimi, Niklas von Danwitz, Hannah Asperger, Julia Layer, Julius Meissner, Christian Thielscher, Franziska Dorn, Nils Lehnen, Joachim L. Schultze, Gabor C. Petzold, Johannes M. Weller, the GSR‐ET Investigators, Anna Alegiani, Jörg Berrouschot, Tobias Boeck‐Behrens, Georg Bohner, Jan Borggrefe, Albrecht Bormann, Michael Braun, Franziska Dorn, Bernd Eckert, Ulrike Ernemann, Marielle Ernst, Jens Fiehler, Klaus Gröschel, Jörg Hattingen, Gerhard Hamann, Christian Heitkamp, Karl‐Heinz Henn, Fee Keil, Lars Kellert, Hannes Leischner, Alexander Ludolph, Ilko Maier, Omid Nikoubashman, Christian Nolte, Martina Petersen, Sven Poli, Gabor C. Petzold, Arno Reich, Joachim Röther, Christian Riedel, Jan Hendrik Schäfer, Maximilian Schell, Peter Schellinger, Eberhard Siebert, Florian Stögbauer, Götz Thomalla, Steffen Tiedt, Christoph Trumm, Timo Uphaus, Silke Wunderlich

**Affiliations:** ^1^ Department of Vascular Neurology University Hospital Bonn Bonn Germany; ^2^ German Center for Neurodegenerative Diseases Bonn Germany; ^3^ Genomics and Immunoregulation, Life & Medical Sciences (LIMES) Institute University of Bonn Bonn Germany; ^4^ Department of Diagnostic and Interventional Neuroradiology University Hospital Bonn Bonn Germany; ^5^ Department of Neurooncology University Hospital Bonn Bonn Germany

## Abstract

**Objective:**

Predicting long‐term functional outcomes shortly after a stroke is challenging, even for experienced neurologists. Therefore, we aimed to evaluate multiple machine learning models and the importance of clinical/radiological parameters to develop a model that balances minimal input data with reliable predictions of long‐term functional independency.

**Methods:**

Our study utilized data from the German Stroke Registry on patients with large anterior vessel occlusion who underwent endovascular treatment. We trained seven machine learning models using 30 parameters from the first day postadmission to predict a modified Ranking Scale of 0–2 at 90 days poststroke. Model performance was assessed using a 20‐fold cross‐validation and one‐sided Wilcoxon rank‐sum tests. Key features were identified through backward feature selection.

**Results:**

We included 7485 individuals with a median age of 75 years and a median NIHSS score at admission of 14 in our analysis. Our Deep Neural Network model demonstrated the best performance among all models including data from 24 h postadmission. Backward feature selection identified the seven most important features to be NIHSS after 24 h, age, modified Ranking Scale after 24 h, premorbid modified Ranking Scale, intracranial hemorrhage within 24 h, intravenous thrombolysis, and NIHSS at admission. Narrowing the Deep Neural Network model's input data to these features preserved the high performance with an AUC of 0.9 (CI: 0.89–0.91).

**Interpretation:**

Our Deep Neural Network model, trained on over 7000 patients, predicts 90‐day functional independence using only seven clinical/radiological features from the first day postadmission, demonstrating both high accuracy and practicality for clinical implementation on stroke units.

## Introduction

Stroke is a leading cause of morbidity and mortality worldwide, accounting for over 100 million disability‐adjusted life years lost annually.^1^ The consequences of stroke extend to both physical and cognitive capabilities, in many cases significantly impairing the patients' ability to engage in daily activities.^2^, ^3^ The extent of the functional impairment is commonly assessed using the modified Rankin Scale (mRS).^4^ Patients and family members, with their crucial role in patient's reintegration into a routine life, frequently ask physicians for prognostication of the patient's future independency. Although trained neurologists possess extensive experience in managing stroke patients—from emergency admission to poststroke follow‐ups—predicting a patient's functional outcome shortly after admission remains challenging. Currently, these predictions are based on a physician's clinical experience, which are recalls of patterns observed in past cases. However, memory‐driven predictions may lack the precision and objectivity offered by data‐driven approaches, due to different reasons such as cognitive biases.^5^, ^6^

Over the past decade, life sciences have made significant advancements by establishing registries containing large datasets.^7^ An advanced approach of analysis is the application of machine learning (ML) methods, which enable the identification of patterns imperceptible to humans and the leverage of these patterns for predictive purposes.^8^ Different ML models can be utilized, including widely employed regression models, decision trees, and deep neural networks (DNN). The selection of the particular model plays an important role in the accuracy of the prediction.^8^ Most data‐driven predictions of poststroke functional outcomes primarily utilize risk scores or logistic regression methods.^9^ However, a comprehensive evaluation comparing different models for outcome prediction in stroke patients undergoing thrombectomy, including DNNs, and an in‐depth analysis of feature importance, has yet to be conducted.

In light of these considerations, our study was designed to investigate different ML models to determine their efficacy in predicting poststroke functional outcome in the early stroke phase. We intended to develop an ML model with an optimal trade‐off between the minimum number of input data and an adequate prediction of future functional independency according to the 90‐day mRS score in patients with anterior cerebral circulation occlusions undergoing thrombectomy.

## Patients and Methods

**Figure 1:**
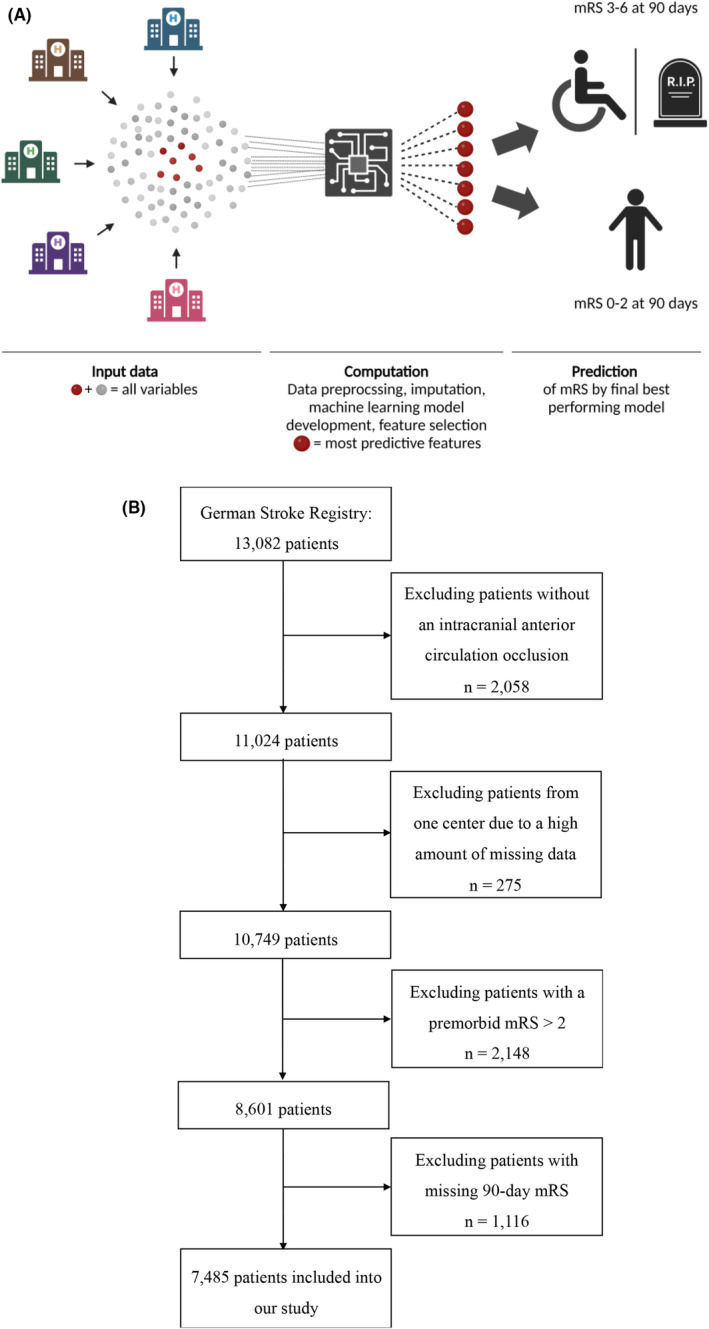
(A) Centralized machine learning approach for the prediction of functional independency at 90 days. (B) Inclusion of patients into our study.

We employed a centralized ML approach encompassing data preprocessing, comparison of input features, selecting major features, and evaluation of different ML models for prediction of functional independency (Fig. 1A). We report our approach in accordance with the TRIPOD+AI guidance for clinical prediction models.^10^

Our study utilized data derived from the German Stroke Registry—Endovascular Treatment (GSR‐ET; ClinicalTrials.gov Identifier: NCT03356392). We used data from patients enrolled from May 2015 until December 2021. The GSR‐ET is an ongoing, academic‐led, open‐label, multicenter initiative that includes patients with intracranial large vessel occlusion strokes (LVOS) undergoing endovascular treatment (ET). Inclusion criteria of the GSR‐ET are as follows: (1) diagnosis of acute ischemic stroke, (2) large vessel occlusion, (3) attempted ET, and (4) age ≥18 years. For our analysis, we included patients with an intracranial anterior circulation occlusion and premorbid functional independence (mRS 0–2, Fig. 1B). This ensured that the prediction models were not biased toward easily anticipated outcomes that are poor regardless of the current stroke event. One study center was excluded due to a high amount of missing data. Furthermore, patients with a missing 90‐day mRS were excluded (Fig. 1B). Our research is compliant with the Declaration of Helsinki's ethical standards. The GSR‐ET has obtained centralized approval from the institutional review board (IRB) at Ludwig‐Maximilian's University Munich (IRB number 689‐15), in addition to necessary approvals from local IRBs. Previous publications have detailed the methods used in the GSR‐ET.^11^ Consent for participation in the registry was obtained from patients or their relatives, as previously described.^12^

The endpoint of our prediction models was the functional outcome measured by the mRS 90 days after stroke. We employed the mRS 90 as a dichotomous endpoint. We separated our study cohort in patients with mRS scores from 0 to 2, denoting functional independency, and scores from 3 to 6, indicating functional dependency and mortality. The input features for prediction covered a range of 30 parameters recorded until the first day after admission (Table S1). Numeric features were standardized, categorial variables were encoded, and missing values in both numeric and categorical variables were imputed using iterative methods, ensuring a complete dataset for analysis. The imputation was based on a Decision Tree Regressor as estimator and on data available until 24 h after admission (Table S1).

Uniform Manifold Approximation and Projections (UMAPs) were used for unsupervised clustering of data. We calculated correlations between the clinical/radiological parameter and the UMAP components by Spearman's rank correlation coefficient. The applied ML models included DNN, Logistic Regression, k‐Nearest Neighbors, XGBoost, Random Forest, Decision Trees, and Support Vector Machines. To evaluate the performance of these models, we utilized a 20‐fold cross‐validation method, employing following metrics with 95% confidence intervals: accuracy, recall, area under the curve (AUC), precision, and F1‐score. Hyperparameter tuning was performed within the cross‐validation. For the optimization of the DNN's hyperparameters (number of input neurons, number of layers, learning rate, L1‐regularization), we utilized Keras Tuner. We statistically evaluated differences in AUC performance between the highest‐performing model (DNN) and other models using a one‐sided Wilcoxon rank‐sum test.^13^ Statistical significance was accepted at *p* < 0.05.

Feature analysis was executed on the DNN model, Logistic Regression model, k‐Nearest Neighbors model, XGBoost model, and Decision Trees model. Built‐in feature importance, based on decrease in node impurity, was utilized for Decision Trees, Random Forest, and XGBoost models. Logistic Regression's feature impact was assessed through coefficient calculations. For the DNN model, permutation feature importance was applied. For the feature selection process, we implemented a backward feature elimination strategy on our best‐performing model (DNN). To assess the performance of the model with the best trade‐off between number of features and prediction quality, we reserved 20% of our dataset for final testing. This ensured that our final testing was conducted on data that was not used during the feature selection process. The remaining 80% of the dataset underwent a split of 80% for training/validation (80%/20%) and 20% for interim testing of each DNN model with a different set of input features. In each iteration of the backward feature selection, we trained a new DNN model, removing the least significant feature from the previous model. For final testing, the optimal model was trained on the entire 80% portion of the dataset designated for model development and was evaluated using the 20% reserved test set to assess its performance.

Median and first (Q1) as well as third (Q3) quartile were utilized for non‐normally distributed data, whereas mean and standard deviation (SD) were used for normally distributed data. The distribution of data was assessed through histograms or statistically.

All analyses were conducted with Python, version 3.11.5, within a Jupyter Notebook environment, version 6.5.4. We employed ChatGPT 4 (OpenAI) as assisting tool for coding and language editing.

## Results

### Characteristics of the study cohort

A total of 7485 patients from 27 study centers with a median age of 75 years were included into our study (Table S2). Half of the study cohort comprised females. A median Alberta Stroke Program Early CT Score (ASPECTS) of 9 indicated that the extent of early ischemic changes was small upon admission, despite a relatively high National Institutes of Health Stroke Scale (NIHSS) score of 14; emphasizing that the patients were suitable candidates for ET. Our inclusion criteria focused on patients with intracranial LVOS of the anterior circulation; however, we observed that 1% of the patients concurrently exhibited an occlusion of an artery of the posterior circulation. The middle cerebral artery was the most occluded artery (82.4%). Cardioembolism was the main cause of stroke (50.9% of patients). Notably, 41.5% of the patients were initially admitted to hospitals lacking neurointerventional facilities. Regarding cardiovascular risk factors, atrial hypertension emerged as the most prevalent one, affecting 75.6% of the cohort.

**Figure 2:**
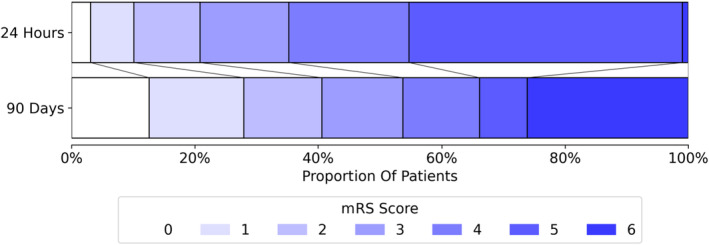
Functional outcome as measured by the modified Rankin Scale (mRS) after 24 h and at 90‐day follow‐up.

Half the study cohort received intravenous thrombolysis (IVT) in addition to ET. A successful outcome of the ET (modified Thrombolysis in Cerebral Infarction scale [mTICI] ≥ 2b) was achieved in 86.1% of patients, requiring a median of two treatment passes. 14.7% of all patients developed an intracranial hemorrhage within 24 h of admission. Only 28.9% of the population was discharged home after stroke, with the remainder being transferred to neurorehabilitation, nursing homes, or other hospitals. In terms of recovery, 40.7% of the patients attained a mRS score of 0–2 after 90 days (Fig. 2). Within 3 months, 26.2% of the patients died. Further detailed patients characteristics are provided in Table S2.

### Unsupervised learning

**Figure 3:**
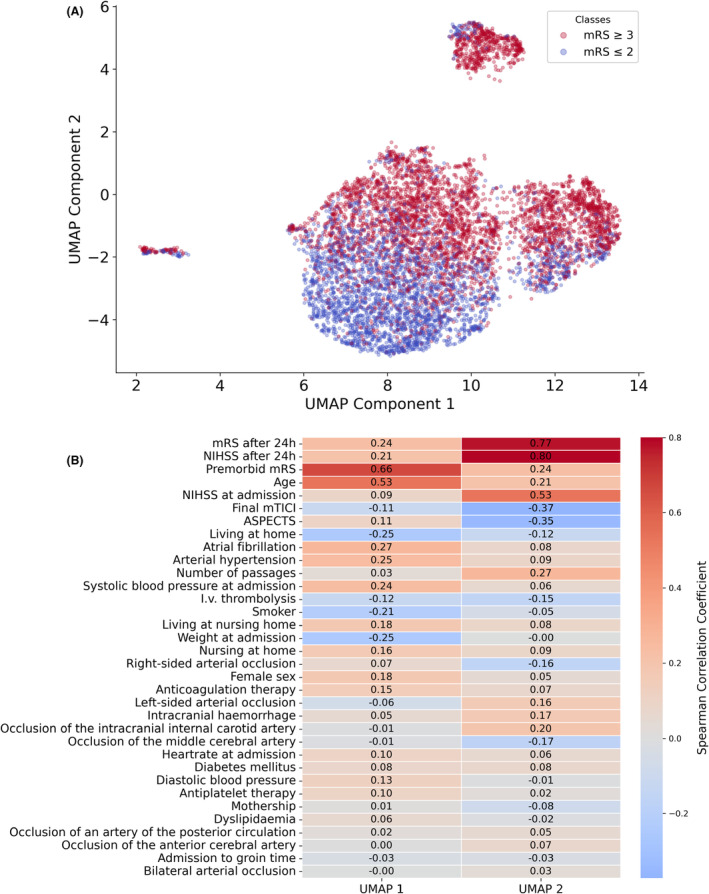
(A) UMAP of input features, labeled according to functional outcome at 90 days poststroke. (B) Correlation matrix for input features and both UMAP components (UMAP 1 and UMAP 2).

A UMAP analysis was performed to reduce the dimensionality of the input data (Fig. 3A). The UMAP components separated most patients with a mRS score of 0–2 at 90 days poststroke from those with a score of ≥3. Further analysis through a correlation matrix identified several key features that strongly impacted the UMAP components (Fig. 3B).

These included the mRS score 24 h postadmission, NIHSS 24 h postadmission, premorbid mRS, age, NIHSS at admission, final mTICI score, and ASPECTS. These features captured relevant predictive information inherent of this dataset. The UMAP component 2, mainly driven by the mRS after 24 h, NIHSS after 24 h, and NIHSS at admission, contributed even more to the prediction of functional outcome. Combining the finding that UMAP components were able to distinguish future functional independency from functional dependency/mortality and which key features contributed most two both components, it is suggested that these key factors may be proficient for a prediction model.

### Supervised learning

We conducted a 20‐fold cross‐validation on seven distinct ML models utilizing all input data. The models included DNN, Logistic Regression, k‐Nearest Neighbors, XGBoost, Random Forest, Decision Tree, and Support Vector Machine.

**Figure 4:**
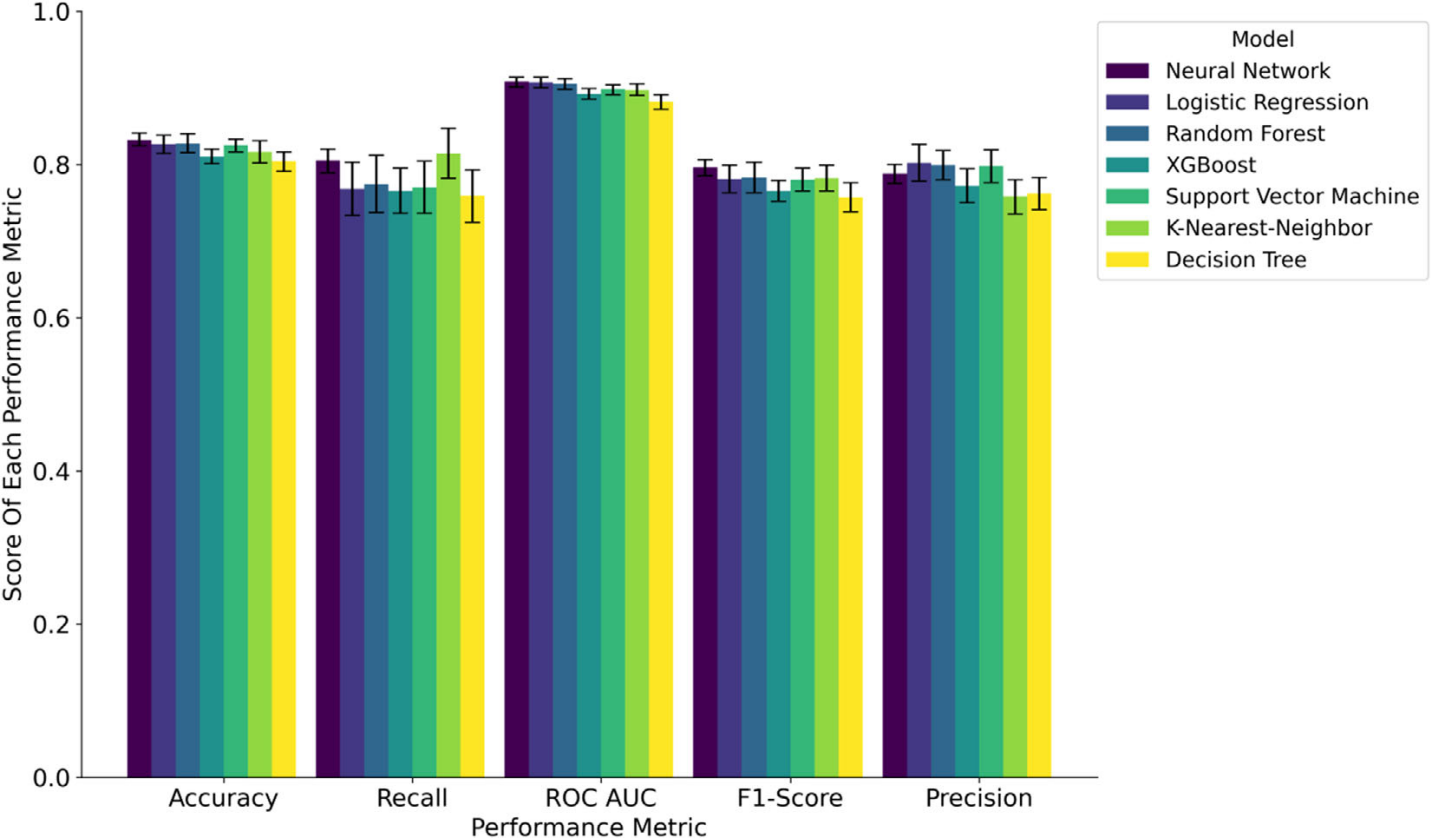
Comparison of performance metrics 24 h post‐treatment across all models.

We first evaluated the models' performance improvements by sequentially incorporating data up to different time points during the early treatment phase. Our analysis began with data exclusively available at the time of admission (Table S3), including imaging parameters and IVT. Subsequently, we extended our analysis to data available up to the ET procedure (Table S4). Finally, we leveraged information available up to 24 h postadmission (Table S5). This methodological approach revealed that each additional timepoint contributed valuable insights on future functional outcome, thereby enhancing all models' performance with increasing AUCs. Notably, the DNN with data collected 24 h postadmission emerged as the best predictive model in almost all performance metrics, with an AUC of 0.908 (CI: 0.901–0.914) (Fig. 4, Table S5), closely followed by the Logistic Regression model (AUC = 0.907 (CI: 0.900–0.914)) and Random Forest model (AUC = 0.905 (CI: 0.898–0.912)). The AUC of the DNN model outperformed the remaining models (XGBoost, Support Vector Machine, k‐Nearest Neighbors, and Decision Tree, all *p* < 0.05, Table S5).

Furthermore, we compared the models' performances at 24 h after admission with a complete case set (*n* = 3184) to investigate the effect of the imputation on the performances (Table S6). All models demonstrated higher performance metrics when applied to the dataset containing imputed data, presenting a positive impact of imputation on model efficacy. The DNN demonstrated again the best performance regarding AUC.

**Figure 5:**
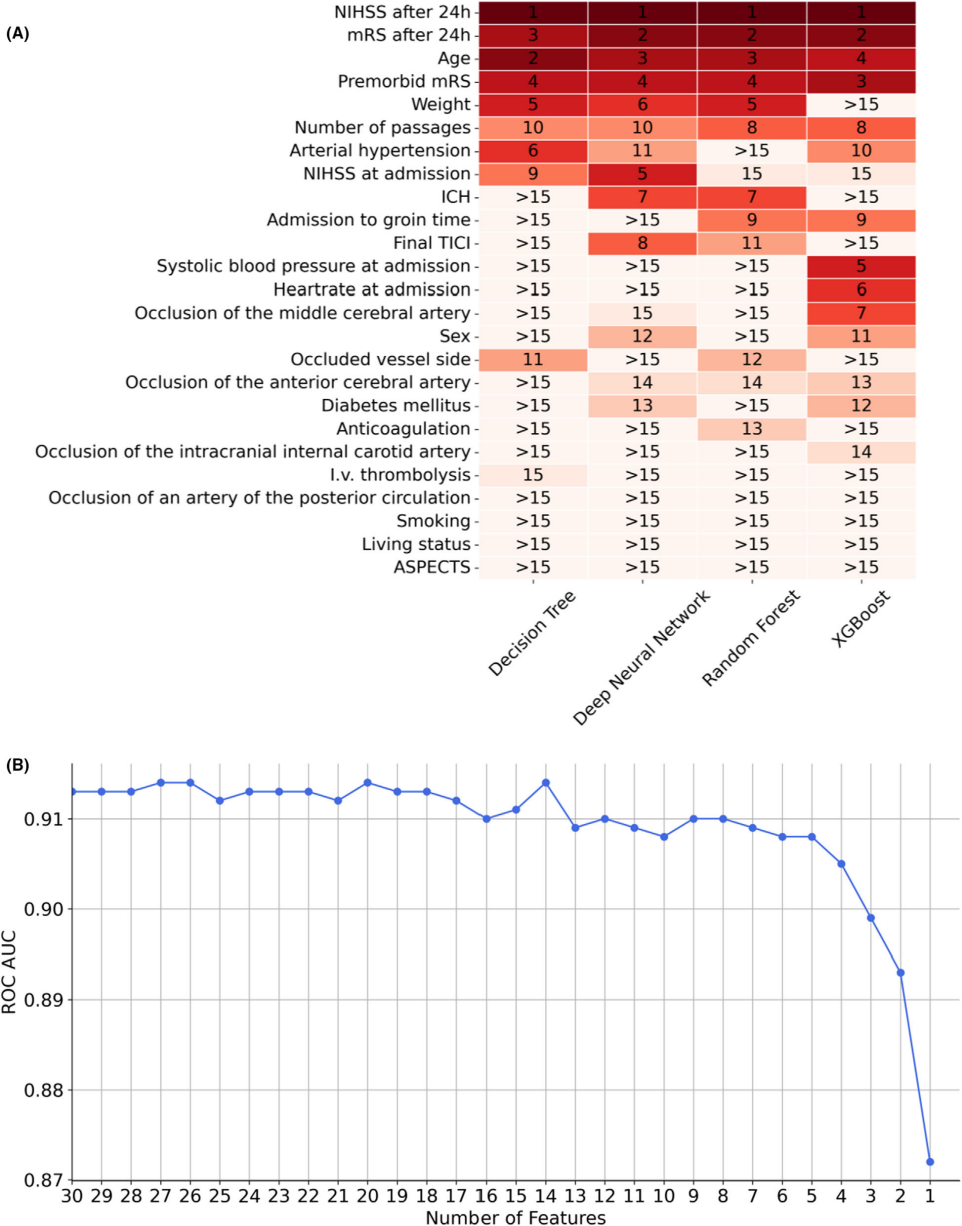
Feature analyses. (A) Comparison of features that ranked among the top 15 in importance across the models. (B) AUC of each deep neural network model trained within the backward feature selection pipeline.

Following the comparison of performance metrics, we examined the impact of features on DNN, Logistic Regression, XGBoost, Random Forest, and Decision Trees. On one hand, we analyzed the feature importance within each model individually (Fig. S1); on the other hand, we compared the features that ranked among the top 15 in importance across the models (Fig. 5A). The most influential factor for prediction was the NIHSS after 24 h, demonstrating the highest impact among all features in each model. It was followed by the mRS after 24 h, which ranked as the second most important factor in every model, followed by age. Generally, numerical parameters had a greater impact than categorical parameters. Among categorical factors, the occurrence of intracranial hemorrhage within 24 h emerged as the most important.

We next aimed to optimize the tradeoff between performance and number of input features. We decided to utilize the DNN model for this purpose since it offers several advantages: It consistently demonstrated high performance in our datasets across various time points; it exhibited the highest performance on the complete dataset, indicating less reliance on imputed data compared to other models; its architecture enables effective extraction of information from high‐dimensional datasets; and it is compatible with learning frameworks such as swarm learning.^13^ We investigated the performance of the DNN with data at 24 h after admission, since it had achieved best performances with this dataset. We applied a backward feature selection (Fig. 5B), that is, an iterative process, each time removing the least influential feature from the model. The performance metrics and weakest features of each model are given in Table S7. Analyzing the AUC performance across different numbers of selected features indicated that using seven features provides a good balance between the number of input data and model performance. The features with the highest impact were, in descending order, NIHSS after 24 h, age, mRS after 24 h, premorbid mRS, intracranial hemorrhage, IVT, and NIHSS at admission. Reducing the feature count below five compromised the performance.

**Figure 6:**
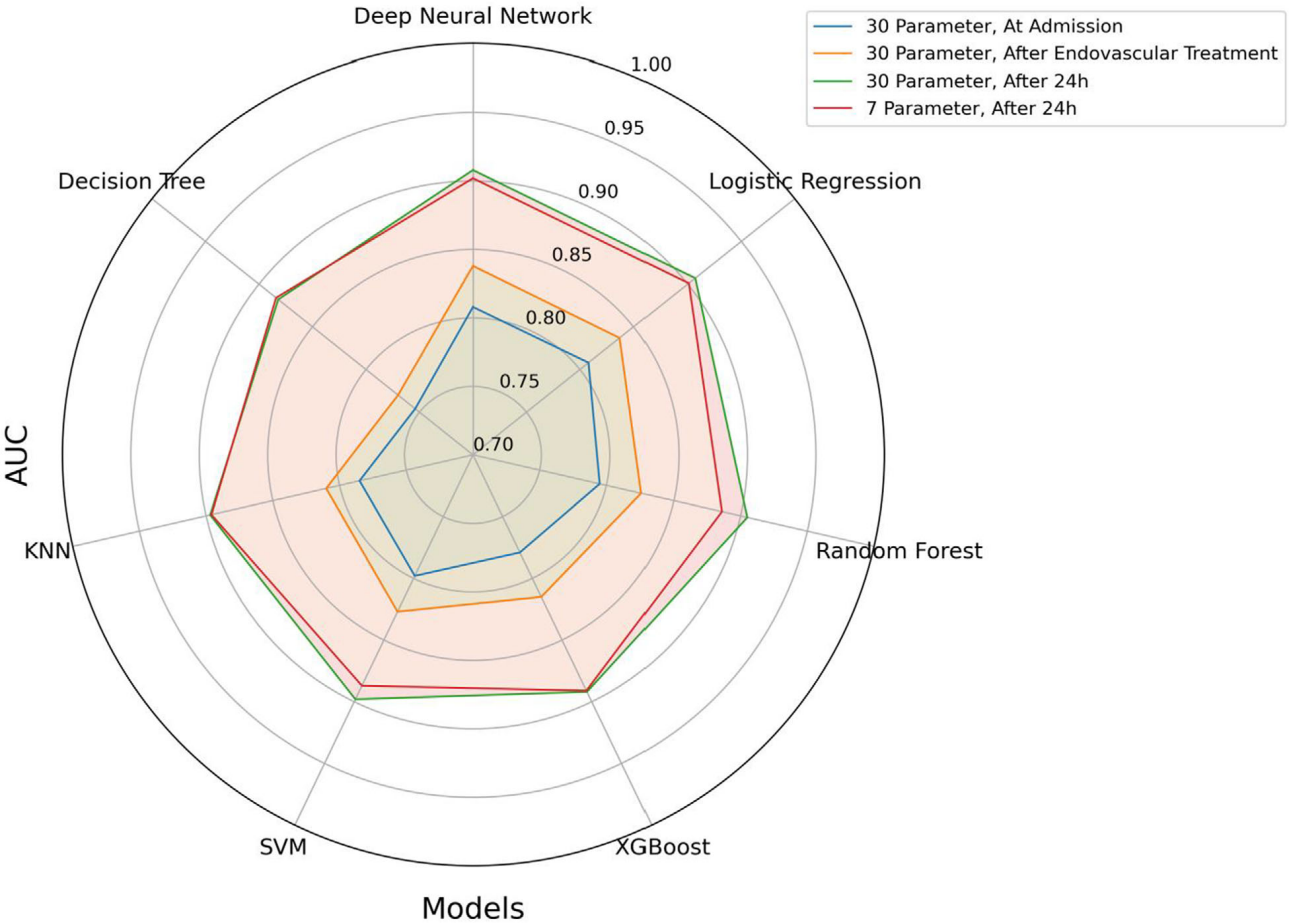
Spider plot of AUCs from all models in key scenarios.

Prior to conducting the backward feature selection, we reserved 20% of the data as an independent test set. We used this dataset for a final testing of the DNN model utilizing the 7 most important features, receiving an accuracy of 0.826, recall of 0.782, *F*1‐Score of 0.782, ROC AUC of 0.893, and precision of 0.782. We next compared all ML models with only these 7 features using 20‐fold cross‐validation (Table S8). The DNN model showed the best performance metrics, with an AUC of 0.902 (CI: 0.894–0.909), closely followed by the Logistic Regression model (AUC = 0.901 (CI: 0.892–0.910)) and KNN (AUC = 0.896 (CI: 0.888–0.905)). The AUC of the DNN model outperformed the remaining models (Random Forest, XGBoost, Support Vector Machine, and Decision Tree) with a *p*‐value <0.05. Additional information including the odds for the logistic regression model was provided in the supplementary materials (Table S9 and Formula S1). Figure 6 summarizes the AUC values obtained from all models across the examined key scenarios.

A recently published mRS prediction model included patients with a premorbid mRS score of >2, while we excluded them from our analysis.^14^ For comparison, we sought to explore the performance of our models upon the inclusion of these patients (total *n* = 8853, Table S10). The models' performances were increased by including patients with a premorbid mRS score of >2. However, including these patients is questionable, as functional independence in patients with premorbid functional dependency is not expected and its prediction seems to be of limited relevance.

## Discussion

Assessing long‐term functional independence early after stroke presents a significant challenge but is crucial for various stakeholders, including patients, their families, and physicians. While models predicting poststroke outcomes currently rely on risk scores or logistic regression techniques,^9^ a systematic comparison of different predictive models, including DNNs, along with an analysis of feature importance as conducted in our study, has not previously been undertaken. Using a large real‐world dataset from over 7000 patients with anterior circulation LVOS undergoing ET, we performed a comparative analysis involving seven different ML models based on 30 clinical parameters and radiological findings reported within the first 24 h postadmission for prediction of long‐term functional outcome. Our findings demonstrate the robust quality of a DNN model in predicting the mRS at 90 days poststroke as a binary endpoint across various scenarios, including different time points, varying numbers of input data, and both with and without imputation of missing data. Achieving an AUC of up to 0.915 (CI: 0.909–0.921) (Table S10), the DNN model emerges as a viable candidate for implementation in clinical practice.

Based on a backward feature selection, the DNN model was simplified by minimizing the number of input features to seven while maintaining robust performance metrics. These features were NIHSS at admission and after 24 h, age, mRS after 24 h, premorbid mRS, intracranial hemorrhage within 24 h, and IVT. Most of these features were critical for differentiating the mRS in both unsupervised and supervised learning approaches and ranked high in feature importance analyses across all models, underscoring their robust value in providing information across various mathematical algorithms. Intriguingly, minimizing the initial 30 input features to these seven key variables had only a minor impact on the performance of the DNN model, reducing the initial AUC of 0.908 (CI: 0.901–0.914) (Table S5) to an AUC of 0.902 (CI: 0.894–0.909) (Table S8). This observation suggests that the 30 features, including clinically considered important data such as preexisting medical conditions or medications, do not add additional relevant predictive information beyond what is captured by the selected seven features. Interestingly, apart from NIHSS after 24 h, the mRS after 24 h also enriched the model, even though both scores mirror functional outcome. While NIHSS primarily provides an assessment of specific neurological deficits, predominantly associated with infarctions in the left territory of the middle cerebral artery,^15^, ^16^ the mRS broadly evaluates the patient's ability to perform daily physical activities such as self‐care and walking.^4^ This distinction underscores the complementary nature of the information provided by NIHSS and mRS to our model. Both scores have a strong impact on predicting long‐term functional independence. This is likely not only because they reflect short‐term functional outcome but also because they represent a composite of symptoms instead of a single distinct symptom, thereby increasing the amount of provided information. In addition to these scores, age, IVT, and intracranial hemorrhage were of importance for the performance of the reduced DNN model. These parameters reflect main information about stroke patients' trajectories such as time to acute care (IVT), complications (intracranial hemorrhage), and rehabilitation potential (age).

By comparing models at three different time points (at admission, immediately after ET, and 24 h after admission), we demonstrated that optimal predictions are obtained using data up to 24 h postadmission, hereby including the acute phase of the disease and surpassing ML models restricted to earlier time points.^17^ This approach aligns with research by Chalos et al., who indicated that including post‐treatment data from the following day significantly enhanced their regression model for the prediction of mRS after stroke.^14^ Their proposed model utilized nine parameters including brain collateralization assessed by CT‐angiography, which might not routinely be available at all centers.^14^ As illustrated by the similar performance metrics of our model, it is suggested that robust outcome prediction is already possible with less and easier accessible variables. Further improvements could potentially be achieved by integrating additional data layers, such as molecular multi‐omics or deep clinical/radiological phenotyping.^18^, ^19^, ^20^ Of note, the mRS is subject to inter‐observer variability, which may limit the maximal achievable performance of prediction models.^4^ Therefore, the current benchmark of technical performance might approach its upper limit near an AUC of 0.92.^20^

A limitation of our study was the absence of an external validation cohort. Instead, we employed a 20‐fold internal cross‐validation method, which is as robust method for validation.^21^ The retrospective nature of our analyses, limited to study centers from one country (Germany), coupled with missing data in the GSR‐ET dataset, represents additional constraints. Future studies should further validate the models on comprehensive datasets from international cohorts.

Assessing the impact of 30 clinical and radiological features evaluated during the first 24 h across seven ML models, we developed a ML model based on DNN for the prediction of functional independence at 90‐day follow‐up in acute stroke patients undergoing ET. This model, which utilized data from over 7000 patients, includes seven relevant and easily accessible features—NIHSS after 24 h, age, mRS after 24 h, premorbid mRS, intracranial hemorrhage within 24 h, IVT, and NIHSS at admission. Achieving an AUC of 0.9 (CI: 0.89–0.91), its high predictive performance coupled with the simplicity of its feature set makes this DNN model an ideal candidate for implementation in clinical routine.

## Author Contributions

O.S.S. and J.M.W. conceptualized and designed the study; O.S.S., F.J.B., L.N., S.S., T.E., N.V.D., H.A., J.L., J.M., F.D., N.L., G.C.P., C.T., and J.M.W. acquired and analyzed data; OSS and JMW wrote the manuscript with input from all other authors. All authors approved the final version of the manuscript.

## Funding Information

This research received no specific grant from any funding agency in the public, commercial, or not‐for‐profit sectors.

## Conflicts of Interest

The authors declare that there is no conflict of interest.

## Informed Consent

Consent for participation in the registry was obtained from patients or their relatives, as previously described.^12^

## Trial Registration

ClinicalTrials.gov NCT03356392.

## Supporting information


Data S1.

## Data Availability

The raw data are subject to the General Data Protection Regulation of the European Union and can be requested from the German Stroke Registry Steering Committee.

## References

[1] Avan A , Digaleh H , Di Napoli M , et al. Socioeconomic status and stroke incidence, prevalence, mortality, and worldwide burden: an ecological analysis from the global burden of disease study 2017. BMC Med. 2019;17(1):191. 31647003 10.1186/s12916-019-1397-3 PMC6813111

[2] Sun J‐H , Tan L , Yu J‐T . Post‐stroke cognitive impairment: epidemiology, mechanisms and management. Ann Transl Med. 2014;2(8):80. 25333055 10.3978/j.issn.2305-5839.2014.08.05 PMC4200648

[3] Hankey GJ , Jamrozik K , Broadhurst RJ , Forbes S , Anderson CS . Long‐term disability after first‐ever stroke and related prognostic factors in the Perth community stroke study, 1989–1990. Stroke. 2002;33(4):1034‐1040. 11935057 10.1161/01.str.0000012515.66889.24

[4] Broderick JP , Adeoye O , Elm J . Evolution of the modified Rankin scale and its use in future stroke trials. Stroke. 2017;48(7):2007‐2012. 28626052 10.1161/STROKEAHA.117.017866 PMC5552200

[5] Saposnik G , Redelmeier D , Ruff CC , Tobler PN . Cognitive biases associated with medical decisions: a systematic review. BMC Med Inform Decis Mak. 2016;16(1):138. 27809908 10.1186/s12911-016-0377-1 PMC5093937

[6] O'Sullivan ED , Schofield SJ . Cognitive bias in clinical medicine. J R Coll Physicians Edinb. 2018;48(3):225‐232. 30191910 10.4997/JRCPE.2018.306

[7] Piovani D , Bonovas S . Real world‐big data analytics in healthcare. Int J Environ Res Public Health. 2022;19(18):11677. 36141962 10.3390/ijerph191811677 PMC9517048

[8] Purushotham S , Meng C , Che Z , Liu Y . Benchmarking deep learning models on large healthcare datasets. J Biomed Inform. 2018;83:112‐134. 29879470 10.1016/j.jbi.2018.04.007

[9] Kremers F , Venema E , Duvekot M , et al. Outcome prediction models for endovascular treatment of ischemic stroke: systematic review and external validation. Stroke. 2022;53(3):825‐836. 34732070 10.1161/STROKEAHA.120.033445 PMC8884132

[10] Collins GS , Moons KGM , Dhiman P , et al. TRIPOD+AI statement: updated guidance for reporting clinical prediction models that use regression or machine learning methods. BMJ. 2024;385:e078378. 38626948 10.1136/bmj-2023-078378 PMC11019967

[11] Alegiani AC , Dorn F , Herzberg M , et al. Systematic evaluation of stroke thrombectomy in clinical practice: the German stroke registry endovascular treatment. Int J Stroke. 2019;14(4):372‐380. 30346260 10.1177/1747493018806199

[12] Schaefer JH , Kurka N , Keil F , et al. Endovascular treatment for ischemic stroke with the drip‐and‐ship model‐insights from the German stroke registry. Front Neurol. 2022;13:973095. 36081874 10.3389/fneur.2022.973095 PMC9445809

[13] Warnat‐Herresthal S , Schultze H , Shastry KL , et al. Swarm learning for decentralized and confidential clinical machine learning. Nature. 2021;594(7862):265‐270. 34040261 10.1038/s41586-021-03583-3 PMC8189907

[14] Chalos V , Venema E , Mulder MJHL , et al. Development and validation of a postprocedural model to predict outcome after endovascular treatment for ischemic stroke. JAMA Neurol. 2023;80(9):940‐948. 37523199 10.1001/jamaneurol.2023.2392 PMC10391355

[15] Lyden P . Using the National Institutes of Health stroke scale: a cautionary tale. Stroke. 2017;48(2):513‐519. 28077454 10.1161/STROKEAHA.116.015434

[16] Vitti E , Kim G , Stockbridge MD , Hillis AE , Faria AV . Left hemisphere bias of NIH stroke scale is most severe for middle cerebral artery strokes. Front Neurol. 2022;13:912782. 35775058 10.3389/fneur.2022.912782 PMC9237381

[17] Quandt F , Flottmann F , Madai VI , et al. Machine learning‐based identification of target groups for thrombectomy in acute stroke. Transl Stroke Res. 2023;14(3):311‐321. 35670996 10.1007/s12975-022-01040-5 PMC10159968

[18] Bonaguro L , Schulte‐Schrepping J , Ulas T , Aschenbrenner AC , Beyer M , Schultze JL . A guide to systems‐level immunomics. Nat Immunol. 2022;23(10):1412‐1423. 36138185 10.1038/s41590-022-01309-9

[19] Lin Y‐H , Chung C‐T , Chen C‐H , et al. Association of temporalis muscle thickness with functional outcomes in patients undergoing endovascular thrombectomy. Eur J Radiol. 2023;163:110808. 37080063 10.1016/j.ejrad.2023.110808

[20] Liu Y , Yu Y , Ouyang J , et al. Functional outcome prediction in acute ischemic stroke using a fused imaging and clinical deep learning model. Stroke. 2023;54(9):2316‐2327. 37485663 10.1161/STROKEAHA.123.044072 PMC11229702

[21] Collins GS , Dhiman P , Ma J , et al. Evaluation of clinical prediction models (part 1): from development to external validation. BMJ. 2024;384:e074819. 38191193 10.1136/bmj-2023-074819 PMC10772854

