# Supplementary material for: Machine learning models for outcome prediction in thrombectomy for large anterior vessel occlusion
Source: Ann Clin Transl Neurol. 2024 Aug 23;11(10):2696–706. doi: 10.1002/acn3.52185 (PMC11514938; doi:10.1002/acn3.52185)
Supplement: Supplementary file 1 — Data S1. [file ACN3-11-2696-s001.docx]

**Machine learning models for outcome prediction in thrombectomy for large anterior vessel occlusion**

**Supplemental material**

**Supplementary Tables:**

**Supplementary Table 1**: Parameter for training of machine learning models.

| **Parameter at admission** |
| --- |
| Age |
| Sex |
| Living status |
| NIHSS at admission |
| Premorbid modified Rankin Scale score |
| Systolic blood pressure at admission |
| Diastolic blood pressure at admission |
| Heart rate at admission |
| Weight at admission |
| Known diabetes mellitus at admission |
| Known arterial hypertension at admission |
| Known dyslipidemia at admission |
| Known atrial fibrillation at admission |
| Active smoking |
| Antiplatelet therapy |
| Anticoagulant therapy |
| Initial admission to a non-interventional hospital |
| Occluded vessel side |
| Occlusion of the intracranial internal carotid artery |
| Occlusion of the middle cerebral artery |
| Occlusion of the anterior cerebral artery |
| Occlusion of an artery of the posterior circulation |
| ASPECTS at admission |
| Intravenous thrombolysis |
| **Additional parameter after endovascular therapy** |
| Time for admission to groin puncture |
| Number of passages during endovascular treatment |
| Final modified Thrombolysis in Cerebral Infarction score |
| **Parameter 24h after admission** |
| NIHSS at 24h |
| Modified Rankin Scale score at 24h |
| Intracranial hemorrhage within 24h |

ASPECTS = Alberta stroke programme early CT score, NIHSS = National Institutes of Health Stroke Scale.

**Supplementary Table 2**: Baseline characteristics as well as therapy and outcome parameter.

| **Baseline characteristics** | |
| --- | --- |
| Age (years), median (Q1-Q3) | 75 (64-82) |
| Sex (female), n (%) | 3,769 (50.4%) |
| Patient weight (kg), mean (SD) | 79 (17) |
| Diabetes mellitus, n (%) | 1,598 (21.4%) |
| Arterial hypertension, n (%) | 5,638 (75.6%) |
| Dyslipidemia, n (%) | 3,113 (41.8%) |
| Atrial fibrillation, n (%) | 3,001 (40.3%) |
| Smoking, n (%) | 1,215 (17.2%) |
| Living status |  |
| - At home n (%) | - 6,995 (94.3%) |
| - Nursing at home n (%) | - 228 (3.1%) |
| - Nursing home n (%) | - 195 (2.6%) |
| Premorbid modified Rankin Scale Score, median (Q1-Q3) | 0 (0-1) |
| - mRS = 0, n (%) | 5,520 (73.7%) |
| - mRS = 1, n (%) | 1,146 (15.3%) |
| - mRS = 2, n (%) | 819 (10.9%) |
| Antiplatelet therapy | 2,162 (29.2%) |
| Anticoagulation | 971 (13.1%) |
| NIHSS, median (Q1-Q3) | 14 (9-18) |
| ASPECTS, median (Q1-Q3) | 9 (7-10) |
| Systolic blood pressure (mmHg), mean, (SD) | 152 (27) |
| Diastolic blood pressure (mmHg), mean (SD) | 83 (17) |
| Heart rate (beats/min), mean (SD) | 82 (19) |
| Occluded vessel side |  |
| - Left, n (%) | 3,889 (52.0%) |
| - Right, n (%) | 3,544 (47.4%) |
| - Bilateral, n (%) | 43 (0.6%) |
| Initial admission to a non-interventional hospital | 3,110 (41.5%) |
| Occlusion of a posterior artery | 75 (1.0%) |
| Occlusion of an anterior artery | 7,485 (100%) |
| - Anterior cerebral artery | 243 (3.2%) |
| - Middle cerebral artery | 6,167 (82.4%) |
| - Intracranial internal carotid artery | 1,839 (24.6%) |
| Stroke aetiology |  |
| - Cardioembolism, n (%) | 3,782 (50.9%) |
| - Large-artery atherosclerosis (embolus / thrombosis), n (%) | 1,797 (24.2%) |
| - Stroke of undetermined aetiology, n (%) | 1,351 (18.2%) |
| - Stroke of other determined aetiology, n (%) | 352 (4.7%) |
| - Dissection, n (%) | 137 (1.8%) |
| - Small-vessel occlusion, n (%) | 6 (0.1%) |
| **Therapy and outcome parameter** | |
| Intravenous thrombolysis, n (%) | 3,761 (50.4%) |
| Time for admission to groin puncture, min, median (Q1-Q3) | 69 (46–97) |
| Number of passages during endovascular treatment, median (Q1-Q3) | 2 (1-3) |
| Final mTICI score ≥ 2b, n (%) | 6,345 (86.1%) |
| Modified Rankin Scale Score after 24h, median (Q1-Q3) | 4 (3-5) |
| NIHSS after 24h, median, (Q1-Q3) | 10 (4-18) |
| Intracranial hemorrhage within 24h, n (%) | 1,101 (14.7%) |
| Duration of hospitalization (days), median (Q1-Q3) | 8 (5-13) |
| Discharge modality |  |
| - Neurorehabilitation, n (%) | 3,354 (52.7%) |
| - Home, n (%) | 1,843 (28.9%) |
| - Hospital, n (%) | 1,049 (16.5%) |
| - Nursing home, n (%) | 124 (1.9%) |
| Discharge NIHSS, median (Q1-Q3) | 5 (1-12) |
| Discharge modified Rankin Scale Score, median (Q1-Q3) | 4 (2-5) |
| In-hospital mortality, n (%) | 1,251 (16.7%) |
| Modified Rankin Scale Score after 90 days, median (Q1-Q3) | 3 (1-6) |

ACA = Anterior Cerebral Artery, ASPECTS = Alberta Stroke Program Early CT Score, MCA = Middle Cerebral Artery, mRS = modified Rankin Scale Score, mTICI = modified Thrombolysis in Cerebral Infarction, NIHSS = National Institutes of Health Stroke Scale, Q1 = First Quartile, Q3 = Third Quartile, SD = Standard Deviation.

**Supplementary Table 3:** Model performances via 20-fold cross validation with dataset at admission.

| **Model** | **Accuracy** | **Recall** | **ROC AUC** | **F1-Score** | **Precision** | **p-value** |
| --- | --- | --- | --- | --- | --- | --- |
| Neural Network | 0.736 (0.727-0.745) | 0.648 (0.632-0.664) | 0.808 (0.797-0.819) | 0.666 (0.654-0.677) | 0.686 (0.673-0.699) | - |
| Logistic Regression | 0.736 (0.726-0.745) | 0.625 (0.603-0.648) | 0.808 (0.796-0.820) | 0.657 (0.643-0.671) | 0.696 (0.680-0.712) | 0.462 |
| Random Forest | 0.727 (0.713-0.740) | 0.602 (0.570-0.635) | 0.795 (0.781-0.808) | 0.640 (0.617-0.662) | 0.687 (0.669-0.705) | 0.083 |
| XGBoost | 0.713 (0.702-0.724) | 0.624 (0.605-0.643) | 0.779 (0.767-0.791) | 0.638 (0.625-0.652) | 0.656 (0.638-0.674) | <0.001 |
| Support Vector Machine | 0.736 (0.725-0.748) | 0.624 (0.600-0.648) | 0.798 (0.786-0.810) | 0.657 (0.640-0.674) | 0.697 (0.680-0.713) | 0.121 |
| k-Nearest Neighbors | 0.715 (0.705-0.725) | 0.612 (0.586-0.637) | 0.785 (0.773-0.798) | 0.634 (0.619-0.650) | 0.663 (0.647-0.678) | 0.015 |
| Decision Tree | 0.706 (0.694-0.719) | 0.597 (0.569-0.626) | 0.754 (0.740-0.769) | 0.622 (0.603-0.641) | 0.653 (0.635-0.670) | <0.001 |

Performance metrics are demonstrated as mean (95% confidence interval); p-value refers to the one-sided Wilcoxon rank sum test comparing the AUC to the best-performing model.

**Supplementary Table 4:** Model performances via 20-fold cross validation with dataset at endovascular treatment.

| **Model** | **Accuracy** | **Recall** | **ROC AUC** | **F1-Score** | **Precision** | **p-value** |
| --- | --- | --- | --- | --- | --- | --- |
| Neural Network | 0.765 (0.755-0.775) | 0.709 (0.688-0.730) | 0.838 (0.828-0.849) | 0.710 (0.695-0.724) | 0.712 (0.700-0.724) | - |
| Logistic Regression | 0.759 (0.749-0.770) | 0.676 (0.651-0.702) | 0.837 (0.826-0.848) | 0.694 (0.679-0.710) | 0.717 (0.701-0.733) | 0.463 |
| Random Forest | 0.750 (0.736-0.763) | 0.623 (0.594-0.652) | 0.826 (0.812-0.839) | 0.668 (0.648-0.688) | 0.725 (0.704-0.745) | 0.051 |
| XGBoost | 0.740 (0.727-0.752) | 0.659 (0.631-0.687) | 0.815 (0.802-0.828) | 0.672 (0.654-0.690) | 0.689 (0.671-0.707) | 0.001 |
| Support Vector Machine | 0.757 (0.746-0.768) | 0.662 (0.640-0.685) | 0.827 (0.816-0.839) | 0.689 (0.673-0.704) | 0.720 (0.702-0.738) | 0.087 |
| k-Nearest Neighbors | 0.731 (0.719-0.743) | 0.761 (0.741-0.781) | 0.810 (0.798-0.822) | 0.697 (0.685-0.708) | 0.644 (0.629-0.659) | <0.001 |
| Decision Tree | 0.708 (0.696-0.720) | 0.630 (0.603-0.656) | 0.770 (0.757-0.784) | 0.636 (0.620-0.652) | 0.647 (0.628-0.665) | <0.001 |

Performance metrics are demonstrated as mean (95% confidence interval); p-value refers to the one-sided Wilcoxon rank sum test comparing the AUC to the best-performing model.

**Supplementary Table 5:** Model performances via 20-fold cross validation with dataset at 24h after admission.

| **Model** | **Accuracy** | **Recall** | **ROC AUC** | **F1-Score** | **Precision** | **p-value** |
| --- | --- | --- | --- | --- | --- | --- |
| Neural Network | 0.832 (0.824-0.841) | 0.805 (0.789-0.820) | 0.908 (0.901-0.914) | 0.796 (0.785-0.806) | 0.788 (0.775-0.800) | - |
| Logistic Regression | 0.826 (0.814-0.838) | 0.768 (0.733-0.803) | 0.907 (0.900-0.914) | 0.781 (0.763-0.799) | 0.802 (0.778-0.826) | 0.505 |
| Random Forest | 0.827 (0.815-0.840) | 0.774 (0.737-0.812) | 0.905 (0.898-0.912) | 0.783 (0.763-0.803) | 0.799 (0.780-0.818) | 0.32 |
| XGBoost | 0.810 (0.801-0.820) | 0.765 (0.736-0.795) | 0.892 (0.885-0.899) | 0.765 (0.752-0.779) | 0.772 (0.750-0.794) | <0.001 |
| Support Vector Machine | 0.825 (0.816-0.833) | 0.770 (0.736-0.804) | 0.898 (0.891-0.904) | 0.780 (0.765-0.795) | 0.798 (0.776-0.819) | 0.025 |
| k-Nearest Neighbors | 0.816 (0.802-0.831) | 0.814 (0.782-0.847) | 0.897 (0.890-0.905) | 0.782 (0.765-0.799) | 0.758 (0.735-0.780) | 0.034 |
| Decision Tree | 0.804 (0.791-0.816) | 0.759 (0.724-0.793) | 0.882 (0.872-0.891) | 0.757 (0.738-0.776) | 0.762 (0.741-0.783) | <0.001 |

Performance metrics are demonstrated as mean (95% confidence interval); p-value refers to the one-sided Wilcoxon rank sum test comparing the AUC to the best-performing model.

**Supplementary Table 6:** Model performances via 20-fold cross validation with dataset at 24h after admission, complete case analysis.

| **Model** | **Accuracy** | **Recall** | **ROC AUC** | **F1-Score** | **Precision** | **p-value** |
| --- | --- | --- | --- | --- | --- | --- |
| Neural Network | 0.794 (0.776-0.812) | 0.800 (0.774-0.825) | 0.870 (0.856-0.883) | 0.786 (0.767-0.805) | 0.774 (0.753-0.796) | - |
| Logistic Regression | 0.789 (0.766-0.812) | 0.766 (0.728-0.804) | 0.862 (0.836-0.888) | 0.773 (0.746-0.800) | 0.784 (0.760-0.809) | 0.463 |
| Random Forest | 0.793 (0.774-0.812) | 0.785 (0.751-0.819) | 0.861 (0.842-0.880) | 0.781 (0.759-0.803) | 0.779 (0.761-0.797) | 0.292 |
| XGBoost | 0.772 (0.750-0.793) | 0.765 (0.729-0.802) | 0.842 (0.822-0.862) | 0.759 (0.733-0.784) | 0.755 (0.735-0.774) | 0.021 |
| Support Vector Machine | 0.786 (0.768-0.805) | 0.778 (0.749-0.807) | 0.854 (0.832-0.876) | 0.774 (0.753-0.795) | 0.773 (0.752-0.793) | 0.155 |
| k-Nearest Neighbors | 0.742 (0.717-0.766) | 0.913 (0.893-0.934) | 0.857 (0.833-0.881) | 0.771 (0.752-0.789) | 0.668 (0.644-0.692) | 0.231 |
| Decision Tree | 0.769 (0.751-0.788) | 0.756 (0.715-0.797) | 0.840 (0.821-0.858) | 0.754 (0.729-0.779) | 0.756 (0.739-0.773) | 0.007 |

Performance metrics are demonstrated as mean (95% confidence interval); p-value refers to the one-sided Wilcoxon rank sum test comparing the AUC to the best-performing model.

**Supplementary Table 7:** Backward feature selection of the deep neuronal network.

| **Number of features** | **Accuracy** | **Recall** | **ROC AUC** | **F1-Score** | **Precision** | **Weakest feature** |
| --- | --- | --- | --- | --- | --- | --- |
| 30 | 0.837 | 0.837 | 0.913 | 0.81 | 0.785 | Smoker |
| 29 | 0.833 | 0.823 | 0.913 | 0.804 | 0.785 | Diastolic blood pressure |
| 28 | 0.839 | 0.843 | 0.913 | 0.813 | 0.785 | Dyslipidaemia |
| 27 | 0.842 | 0.819 | 0.914 | 0.812 | 0.804 | Diabetes mellitus |
| 26 | 0.839 | 0.819 | 0.914 | 0.808 | 0.798 | Mothership |
| 25 | 0.841 | 0.821 | 0.912 | 0.81 | 0.8 | Occlusion of the middle cerebral artery |
| 24 | 0.837 | 0.819 | 0.913 | 0.807 | 0.795 | Arterial hypertension |
| 23 | 0.84 | 0.831 | 0.913 | 0.811 | 0.793 | Occlusion of an artery of the posterior circulation |
| 22 | 0.832 | 0.823 | 0.913 | 0.803 | 0.784 | Number of passages |
| 21 | 0.839 | 0.845 | 0.912 | 0.813 | 0.784 | Atrial fibrillation |
| 20 | 0.835 | 0.811 | 0.914 | 0.803 | 0.795 | ASPECTS |
| 19 | 0.836 | 0.821 | 0.913 | 0.806 | 0.792 | Occlusion of the intracranial internal carotid artery |
| 18 | 0.84 | 0.833 | 0.913 | 0.812 | 0.792 | Sex |
| 17 | 0.838 | 0.821 | 0.912 | 0.808 | 0.795 | Anticoagulation therapy |
| 16 | 0.832 | 0.825 | 0.91 | 0.803 | 0.782 | Occlusion of the anterior cerebral artery |
| 15 | 0.841 | 0.837 | 0.911 | 0.813 | 0.791 | Admission to groin time |
| 14 | 0.838 | 0.829 | 0.914 | 0.809 | 0.791 | Final modified Thrombolysis in Cerebral Infarction score |
| 13 | 0.836 | 0.817 | 0.909 | 0.805 | 0.793 | Systolic blood pressure at admission |
| 12 | 0.839 | 0.813 | 0.91 | 0.807 | 0.802 | arterial occlusion side |
| 11 | 0.839 | 0.819 | 0.909 | 0.808 | 0.798 | Living status |
| 10 | 0.841 | 0.807 | 0.908 | 0.808 | 0.81 | Antiplatelet therapy |
| 9 | 0.84 | 0.821 | 0.91 | 0.81 | 0.798 | Weight at admission |
| 8 | 0.841 | 0.833 | 0.91 | 0.813 | 0.795 | Heartrate at admission |
| 7 | 0.832 | 0.817 | 0.909 | 0.802 | 0.787 | NIHSS at admission |
| 6 | 0.831 | 0.829 | 0.908 | 0.802 | 0.777 | Intravenous thrombolysis |
| 5 | 0.827 | 0.833 | 0.908 | 0.8 | 0.77 | Intracranial hemorrhage |
| 4 | 0.833 | 0.819 | 0.905 | 0.803 | 0.787 | Premorbid mRS |
| 3 | 0.825 | 0.795 | 0.899 | 0.79 | 0.785 | mRS after 24h |
| 2 | 0.814 | 0.787 | 0.893 | 0.778 | 0.77 | Age |
| 1 | 0.804 | 0.787 | 0.872 | 0.769 | 0.752 | NIHSS after 24h |

ASPECTS = Alberta stroke programme early CT score, NIHSS = National Institutes of Health Stroke Scale.

**Supplementary Table** **8:** Models performances with seven selected input features via 20-fold cross validation.

| **Model** | **Accuracy** | **Recall** | **ROC AUC** | **F1-Score** | **Precision** | **p-value** |
| --- | --- | --- | --- | --- | --- | --- |
| Neural Network | 0.827 (0.819-0.835) | 0.798 (0.778-0.818) | 0.902 (0.894-0.909) | 0.789 (0.779-0.800) | 0.783 (0.770-0.796) | - |
| Logistic Regression | 0.825 (0.811-0.839) | 0.768 (0.731-0.804) | 0.901 (0.892-0.910) | 0.780 (0.759-0.800) | 0.799 (0.777-0.821) | 0.495 |
| Random Forest | 0.813 (0.802-0.824) | 0.753 (0.719-0.787) | 0.886 (0.877-0.896) | 0.765 (0.748-0.781) | 0.783 (0.762-0.805) | 0.007 |
| XGBoost | 0.812 (0.801-0.822) | 0.768 (0.734-0.803) | 0.891 (0.882-0.899) | 0.767 (0.751-0.783) | 0.773 (0.753-0.793) | 0.013 |
| Support Vector Machine | 0.826 (0.812-0.841) | 0.776 (0.736-0.816) | 0.887 (0.878-0.897) | 0.782 (0.760-0.805) | 0.796 (0.775-0.818) | 0.012 |
| k-Nearest Neighbors | 0.817 (0.804-0.830) | 0.731 (0.692-0.769) | 0.896 (0.888-0.905) | 0.763 (0.741-0.784) | 0.806 (0.784-0.829) | 0.177 |
| Decision Tree | 0.811 (0.799-0.824) | 0.765 (0.728-0.802) | 0.884 (0.876-0.893) | 0.766 (0.745-0.786) | 0.772 (0.754-0.791) | 0.001 |

Performance metrics are demonstrated as mean (95% confidence interval); p-value refers to the one-sided Wilcoxon rank sum test comparing the AUC to the best-performing model.

**Supplementary Table 9:** Odds ratios of the logistic regression model for functional independency.

| **Parameter** | **Odds ratio (95% confidence interval)** |
| --- | --- |
| Intravenous thrombolysis | 1.522 (1.363-1.700) |
| Intracranial hemorrhage | 0.434 (0.345-0.547) |
| Age | 0.534 (0.494-0.577) |
| NIHSS at admission | 0.825 (0.756-0.900) |
| Premorbid mRS | 0.627 (0.577-0.681) |
| NIHSS after 24h | 0.313 (0.271-0.361) |
| mRS after 24h | 0.444 (0.396-0.498) |

The logistic regression model was trained/tested on the seven selected features. NIHSS = National Institutes of Health Stroke Scale, mRS = modified Rankin Scale.

**Supplementary Table 10:** Model performances via 20-fold cross validation with the seven selected features including patients irrespective of premorbid functional status.

| **Model** | **Accuracy** | **Recall** | **ROC AUC** | **F1-Score** | **Precision** | **p-value** |
| --- | --- | --- | --- | --- | --- | --- |
| Neural Network | 0.848 (0.841-0.856) | 0.792 (0.771-0.813) | 0.915 (0.909-0.921) | 0.786 (0.773-0.798) | 0.781 (0.771-0.790) | - |
| Logistic Regression | 0.845 (0.832-0.857) | 0.753 (0.714-0.791) | 0.912 (0.901-0.924) | 0.772 (0.750-0.793) | 0.799 (0.778-0.820) | 0.611 |
| Random Forest | 0.834 (0.823-0.845) | 0.743 (0.703-0.782) | 0.901 (0.891-0.912) | 0.757 (0.737-0.778) | 0.781 (0.761-0.801) | 0.02 |
| XGBoost | 0.835 (0.822-0.848) | 0.756 (0.716-0.797) | 0.904 (0.893-0.915) | 0.761 (0.738-0.784) | 0.773 (0.754-0.791) | 0.051 |
| Support Vector Machine | 0.846 (0.835-0.858) | 0.758 (0.720-0.796) | 0.895 (0.883-0.907) | 0.775 (0.753-0.796) | 0.798 (0.779-0.817) | <0.001 |
| k-Nearest Neighbors | 0.835 (0.823-0.847) | 0.713 (0.672-0.753) | 0.909 (0.898-0.920) | 0.751 (0.729-0.773) | 0.804 (0.780-0.828) | 0.328 |
| Decision Tree | 0.834 (0.820-0.848) | 0.757 (0.715-0.800) | 0.893 (0.880-0.906) | 0.760 (0.734-0.786) | 0.769 (0.751-0.787) | 0.001 |

Performance metrics are demonstrated as mean (95% confidence interval); p-value refers to the one-sided Wilcoxon rank sum test comparing the AUC to the best-performing model.

**Supplementary Figure:**

**Supplementary Figure 1**: Feature analyses across five machine learning models.

A) Deep Neural Network, B) Decision Tree, C) Random Forrest, D) XGBoost, E) Logistic Regression.

**Supplementary Formula**

$$\log\left( \frac{p}{1 - p} \right)= - 0.796+ 0.420 * Intravenous Thrombolysis - 0.834 * Intracranial Hemorrhage - 0.627 * Age - 0.192 * NIHSS at Admission - 0.467 * Premorbid mRS - 1.163 * NIHSS after 24h - 0.812 * mRS after 24h$$

**Supplementary Formula 1**: Formula of the logistic regression model.
